# Fiber Loop Ringdown — a Time-Domain Sensing Technique for Multi-Function Fiber Optic Sensor Platforms: Current Status and Design Perspectives

**DOI:** 10.3390/s91007595

**Published:** 2009-09-28

**Authors:** Chuji Wang

**Affiliations:** Department of Physics, and the Institute for Clean Energy Technology, Mississippi State University, Starkville, MS, 39759, USA; E-Mail: cw175@msstate.edu; Tel.: +1-662-325-9455; Fax: +1-662-325-8898

**Keywords:** fiber loop ringdown, cavity ringdown spectroscopy, chemical and physical fiber optic sensors, fiber optic sensor networks, multi-function, remote sensing

## Abstract

Fiber loop ringdown (FLRD) utilizes an inexpensive telecommunications light source, a photodiode, and a section of single-mode fiber to form a uniform fiber optic sensor platform for sensing various quantities, such as pressure, temperature, strain, refractive index, chemical species, biological cells, and small volume of fluids. In FLRD, optical losses of a light pulse in a fiber loop induced by changes in a quantity are measured by the light decay time constants. FLRD measures time to detect a quantity; thus, FLRD is referred to as a time-domain sensing technique. FLRD sensors have near real-time response, multi-pass enhanced high-sensitivity, and relatively low cost (i.e., without using an optical spectral analyzer). During the last eight years since the introduction of the original form of fiber ringdown spectroscopy, there has been increasing interest in the FLRD technique in fiber optic sensor developments, and new application potential is being explored. This paper first discusses the challenging issues in development of multi-function, fiber optic sensors or sensor networks using current fiber optic sensor sensing schemes, and then gives a review on current fiber optic sensor development using FLRD technique. Finally, design perspectives on new generation, multi-function, fiber optic sensor platforms using FLRD technique are particularly presented.

## 1. Introduction

Modern industries and manufacturing need novel sensors to reduce cost, improve efficiency, monitor operation environments, assess the health of civil infrastructure, etc. For example, in coal-fired power plants, a multi-functional sensor system is needed to measure gasifier temperature for an optimized operation, to detect the thickness of gasifier refractory liners for early warning of potential cracks to reduce costs associated with unwanted early shut-down/forced unprepared shut-down, and to quantify trace chemical compounds in off-gas emissions. In the U.S., a 1% improvement in operating efficiency gained from a controls-and-sensors retrofit would result in $409 million in annual fuel savings, and a 1% increase in availability of improved control and accurate sensing would result in an additional 5,000 MW of capacity without additional power plants and at a minimal expense [1,2]. In the auto industries, a sensor system is needed to monitor trace chemicals in exhaust emissions and sense engine temperature, mechanical deformation, tire pressure, and in/out driving climate [3]. Carbon sequestration for enhanced oil recovery (injection of CO_2_ into an oil reservoir to push the oil out and store CO_2_ in the reservoir permanently) needs sensors to simultaneously monitor pressure, temperature, and CO_2_ concentration at multiple points in the reservoir and the sensors must be deployable through a deep down-hole steel pipe with an inner diameter of 4–6 inches [4-7]. In civil engineering (dams, bridges, pipelines, etc.), novel sensors are needed to monitor loading history and mechanical fatigue, detect early leakage, access post-storm damage, and identify toxic chemicals in a remote and timely manner [8-17]. Similar needs in many other applications continue to challenge scientists and engineers to create novel sensors and sensor systems [18-20].

Fiber optic sensors (FOS) have been playing an increasingly important role in the sensing community due to their attractive application features, such as low cost, small footprint, light weight, immunity to electromagnetic interference, and ability to cover long distances and be mutiplexible (for multiple function or single function in multiple sensing locations) [21-29]. FOS have been studied over three decades and have involved several rounds of revolutionary changes with advances in light sources, fiber optics, and spectroscopic methods. To date, sensing mechanisms (or transduction principles) of FOS have been well established [15,28,29]. A wide variety of transduction principles has been reported, such as fluorescence- [30-35], absorption- [36-40], interferometric-based transduction for fiber optic chemical sensors [41-43] and mechanical deformation-, thermal expansion-based transduction for fiber optic physical sensors [e.g., Fabry-Perot interferometric (FPI) pressure/temperature sensors, fiber Bragg grating (FBG) temperature sensors] [44-51]. Although new sensing mechanisms are still being pursued, the driving force for future FOS development lies in *novel sensing platforms* and *enhanced performance* [15,28,29]. The former means innovative sensing schemes, low manufacturing cost, and being technically and economically configurable with an existing sensor network. The combination of sensing mechanisms and sensing platforms dictates the sensor performance. Enhancement performance may have three different perspectives. First, enhanced performance of FOS means high sensitivity, high accuracy, high selectivity, and robustness. Significant strides have been made during the last ten years, and most FOS have demonstrated some or all of the four merits. Second, enhanced performance means rapid response, remote control, and fast data transmission. The majority of the on-going research in the field of FOS is focusing on addressing these issues. Third, enhanced performance means the ability to sense multiple parameters, including physical quantities (e.g., pressure, temperature, stress, vibration, velocity, etc.) and chemical quantities (e.g., concentration, identification, pH value, etc.), in a single sensor system while being readily able to be added to/dropped from an existing sensing network with low costs while still fulfilling the requirements of the first two perspectives.

One of the most significant challenges in creating these types of high-performance, multi-function FOS or sensor systems, which is a fundamental constraint in the sensing communities, is that current FOS are primarily based on the detection of a decrease in light intensity Δ*I* (e.g., absorption-based), a wavelength shift Δλ (e.g., FBG-based), or both (e.g., Raman-, fluorescence-based). To date, none of these conventional sensing schemes can be used as a uniform detection scheme to enable a sensor system to simultaneously detect multiple quantities, such as the aforementioned physical and chemical quantities, because these sensing schemes are sensitive to light intensity fluctuations and power losses, which limit the maximum number of sensors in a single sensor system for detection of multiple quantities. Additionally, integrating several different sensing schemes into a single system not only incurs high networking costs due to the requirements of optical amplification and expensive terminal detection equipment, but also makes signal transmission and data acquisition logistically difficult and inefficient.

One potential breakthrough in addressing the challenging issues stated above is to develop a multi-functional fiber optic sensor platform using the recently developed fiber loop ringdown (FLRD) technology (see Section 3) [52-58]. So far, several types of FLRD-based sensors, such as FLRD gas/liquid sensors [53-59], FLRD pressure (P) sensors [59,60], FLRD Bragg grating temperature (T) sensors [61-63], FLRD strain sensors [64-66], FLRD refractive index sensors [67], and FLRD microfluidics sensors [68-69], have been reported during the last eight years. Each individual sensor has demonstrated high sensitivity (several orders of magnitude higher than a conventional FOS if the same sensing mechanism is employed) and high speed of detection gained from the nature of the FLRD technique [52-71]. Most importantly, all of the aforementioned FLRD-based sensors are based on a universal sensing scheme to measure all quantities (P, T, gas concentration, liquid volume, etc.) in time-domain. Specifically, the optical losses of a laser pulse in the fiber loop due to a change of a measurand are measured by the light intensity decay rate (called the ringdown time). FLRD, virtually, measures time to detect a quantity of interest. FLRD is relatively new in spectroscopy and sensing communities; its potential to create new generation of FOS and sensor systems has not much been discussed.

Our research motivation is to address the challenging issues in creating a multi-function, high performance, fiber optic sensor system. This article first gives a review on sensing functionalities, sensing mechanisms, and sensing schemes of current fiber optic sensors, and then describes the principle of the time-domain FLRD technique. After that, the current status of FLRD-based FOS in terms of chemical sensors and physical sensors is reviewed. Finally, the theoretical consideration and design perspectives of multi-function fiber optic sensor platforms using the FLRD technique are presented and four speculative design modules are given to illustrate the concept of new generation, multi-function, fiber optic sensors or sensor networks based on novel sensing platforms.

## 2. Sensing Functionalities, Sensing Mechanisms, and Sensing Schemes of Current FOS

The field of FOS has yielded more than 2,600 publications in the last ten years alone (searches conducted on SciFinder and Web of Science), and it is almost impossible to cover all aspects of FOS in a single exhaustive review. There are many different FOS classifications according to the measured parameters, the topology of a sensor, the transduction principles, and the light parameter modulated. We may also simply categorize FOS into two major groups based on functionality, fiber optic chemical sensors and fiber optic physical sensors. The former includes detection of chemical quantities, such as concentration and identification of chemical species, pH values, etc. [72-76]; the latter includes physical quantities, such as pressure, force, temperature, stress, strain, vibration, velocity, surface roughness, frequency, etc. [77-86]. Due to the diversity of the quantities (measurands or parameters), a variety of sensing mechanisms has been investigated to improve performance on either one or all of the basic merits of sensitivity, selectivity, accuracy, reliability, and robustness. To date, various sensing mechanisms have been established for individual sensors and will remain largely unchanged in next several years [15,28,29].

However, driven by a myriad of needs in demanding applications, novel sensing platforms and enhanced performance have become the driving forces for future FOS development [15,28,29]. Table 1 lists current FOS in terms of sensing functionalities, sensing mechanisms, and sensing schemes. Although Table 1 is not an all-inclusive FOS list (e.g., biological and medical FOS are not included [87-91]), the major types of FOS in terms of sensing schemes are included. It is clear that most chemical sensors are based on a sensing scheme of intensity vs. frequency (or wavelength) using a spectrometer, i.e., an optical spectral analyzer (OSA). For example, FOS for oxygen is based on the quenching effect of oxygen on certain wavelength-dependent fluorophorers [30,31]. FBG temperature sensors are based on the detection scheme of intensity vs. wavelength using an OSA to measure a shift of the Bragg wavelength due to a change in temperature [43,50,51]. These types of sensing schemes can be defined as frequency-domain sensing schemes. The intensity vs. frequency sensing scheme is working well for each individual sensor in most applications, but it has significant limitations in sensor networking because the detection scheme suffers from light power fluctuations and power losses. Optical amplification is usually employed in a network for the maximum number of sensors to be multiplexed; however, the optical amplification often generates amplified spontaneous emission noise (ASE) that leads to a poor signal-to-noise ratio [92-94].

Different from the frequency-domain FOS, time-domain FOS has become an increasingly active area of research [95-97]. One example are the time-domain reflectormetric FBG temperature sensors. By using a time division multiplexing (TDM) method, Wang's group recently demonstrated the capability of multiplexing 1,000 Bragg grating sensors along a single fiber [95]. In this TDM scheme, a narrow laser pulse is coupled into a fiber along which many weak gratings are scribed so that each reflects only an extremely small fraction of the incident power. This allows many successive laser pulses to be reflected back. From the arrival times of these pulses, the sensor locations can be determined. At the same time, the magnitude of the reflected intensity of each pulse gives an indication of the change of temperature (or pressure) or any activity that can result in a shift of the bandwidth curve of the FBG. This type of sensing scheme is universally applicable. However, in the aforementioned sensing scheme, signals from different sensor heads remain to be detected in the form of intensity vs. time. The sensitivity is determined by spectral resolution, the minimum detectable intensity change, and stability of the light source. Strictly speaking, this type of sensing scheme is still a light intensity based detection scheme, and the ultimate sensitivity is limited to the stability of the light intensity.

## 3. Fiber Loop Ringdown — a Universally Applicable Time-Domain Sensing Scheme for FOS

### 3.1. Origin of Fiber Loop Ringdown

**Figure 1.**
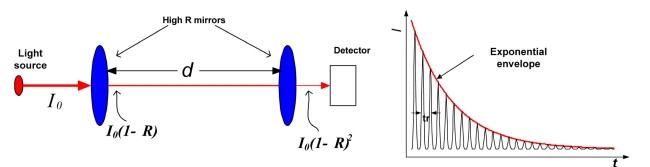
Illustration of the CRDS concept. The presence of additional absorption in the cavity is detected by a shortening of the decay time constant (ringdown time). In the CRDS, the effective absorption path-length is readily increased more than 10,000-fold.

During the last several years, a new fiber loop ringdown (FLRD) technique has been introduced for chemical and physical quantity sensing. The FLRD technique is fundamentally evolved from the well-know cavity ringdown spectroscopy (CRDS) technique [98-106]; CRDS obtains high sensitivities because of the multi-pass nature of the optical absorption path, as illustrated in Figure 1.

Since its introduction, the CRD technique has rapidly developed and matured, from initial applications focusing on weak absorption spectroscopic measurements to now being a fully commercialized process for trace gas analysis and sensing. Although new ideas and the latest technologies have prompted the evolution of the CRDS technique with various forms of ringdown cavities [106-115], all of the CRD techniques are based on one measuring principle: measuring time decay rates (ringdown times) of the light intensity to determine gas species within a gas cell or adsorbed analytes at a surface. This feature has recently been implemented by using a “conceptual cavity” — a fiber loop formed by a section of single mode fiber [52].

### 3.2. The Principle of Fiber Loop Ringdown

A light pulse is coupled into a fiber loop and travels (*rings)* inside the fiber loop for many round trips. In each round trip, a small fraction of the light pulse couples out of the loop into a photodetector through a fiber coupler; and the rest of the light travels in the fiber, experiencing internal fiber transmission losses. The output signal observed by the detector follows an exponential decay. This behavior can be modeled by [59,60]

(1) $$\frac{dI}{dt}=-\frac{IAc}{nL}$$

where *I* is the light intensity at time *t* (we assume the time equals zero when the light source is shut off and a light pulse is injected into the loop), and *L,c, n*, and *A* are the total length of the fiber loop, speed of light in a vacuum, fiber refractive index, and total fiber transmission loss (by percentage) of the light in each round trip, respectively. The total fiber transmission loss includes the fiber absorption loss, the fiber couplersapos;insertion losses, and the fiber scattering loss; and A = *aL* + *E* + *γ*, where αis the wavelength-dependent absorption coefficient for the fiber core material with units of, e.g., cm-1, Eis the total insertion loss of the fiber couplers, and *γ* is the total fiber scattering loss. The solution of Equation (1) describes the temporal decay behavior of the light intensity observed by the detector:

(2) $$I=I_{0}e^{-\frac{c}{nL}At}$$

Equation (2) shows that FLRD measures the light intensity decay rate, not the absolute intensity change, *ΔI*. Therefore, the measurement of *A* is insensitive to fluctuations of *I_0_*, the incident light intensity.

The time required for the light intensity (*I*) to decrease to 1/e of the initial light intensity (*I_0_*), as observed by the detector, is referred to as the ringdown time, τ_0_, and is given by Equation (3a) [59,60]

(3a) $$\tau_{0}=\frac{nL}{cA}$$

(3b) $$\tau =\frac{nL}{c(A+B)}$$

For a given FLRD sensor (pressure, temperature, or strain, etc.), the total transmission loss, *A*, is a constant, which is determined by the physical parameters of the sensor, such as the fiber absorption loss, the couplersapos; insertion losses, the refractive index, and the fiber length. Clearly, the lower the losses of the light in the fiber are, the longer the decay time constants (*τ*_0_) will be. When an external action, such as absorption, or a change of any measurands, such as pressure, temperature, or stress, occurs at one section (sensor head) of the fiber loop, the result is an additional optical loss, *B*, of the light pulse in the fiber loop, which causes a change in the ringdown time, *τ*, given by Equation (3b).

From Equation 3a,b, we have [59,60]:

(4) $$(\frac{1}{\tau }-\frac{1}{\tau_{0}})=\frac{c}{nL}B$$

Equation (4), namely, the principle of FLRD, indicates that for a given fiber ringdown sensor, a change in a sensing activity (e.g., gas absorption, fiber mechanical deformation, thermal expansion, etc.) is determined by measuring *τ*_0_, the ringdown time without the sensing activity, and *τ*, the ringdown time with the activity, and that the term (1/*τ* – 1/*τ*_0_) has a linear relationship with the activity-induced optical loss, *B*.

**Figure 2.**
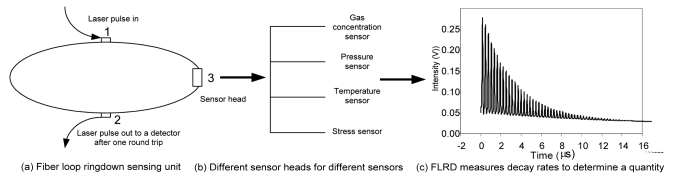
Fiber Loop Ringdown (FLRD) – a universal time-domain sensing scheme.

Figure 2(a) shows a schematic diagram of the universal FLRD sensing scheme (a sensor unit). Figure 2(b) shows that multiple FLRD-based sensors can be fabricated by using different transduction mechanisms for detecting each of the individual measurands. Figure 2(c) shows a typical light intensity decay behavior observed by the photodetector. Each of the separated spikes shows the intensity of the light coming out of the loop after each succesive round trip. The time between two adjacent spikes is the round trip time of the light inside the loop. The envelope follows a single exponential decay. Therefore, the decay rate is immune to pulse-to-pulse light intensity fluctuations. A slower decay rate (longer ringdown time) means lower optical losses of the light in the loop, and vice versa. FLRD measures time to determine a quantity.

### 3.3. Advantages of Fiber Loop Ringdown for FOS

The features of FLRD include: (1) A quantity is measured by measuring the time constant (a time-domain measurement technique); (2) The detection sensitivity is proportionally enhanced by the number of multiple-round trips; (3) The measurement is insensitive to intensity fluctuations of the light source; and (4) An entire ringdown event (the light residence time inside the fiber loop) is fast, e.g., on the order of microseconds, depending on optical losses and fiber length. Furthermore, FLRD also possesses following attributes: (1) FLRD requires low laser power, e.g., ∼μW; (2) FLRD allows FLRD-based multiple sensor units (sensor loops) to be multiplexed due to the uniform sensing scheme and waveguide; (3) Sensor systems (or sensor networks) built on FLRD sensors have no need of optical amplification, thus no associated ASE noise; (4) FLRD offers high configurability, e.g., changing a sensor head in the fiber loop (e.g., from a FBG for temperature sensing to an air-gap for chemical detection) does not necessitate a change of the detectors and their settings because of the uniform time detection scheme; and (5) FLRD sensors, regardless of absorption-based chemical sensors or FBG-FLRD temperature sensors, allow an inexpensive photodiode, instead of an expensive OSA, to be used as a detector. A FLRD sensor system will have low cost as compared to a typical FOS employing an OSA as the detector. As an example, a side-by-side comparison of a time-domain FBG-FLRD temperature sensor with a frequency-domain FBG-OSA temperature sensor can be seen in Table 2.

## 4. Current FLRD-Based FOS

### 4.1. FLRD Chemical Sensors

**Figure 3.**
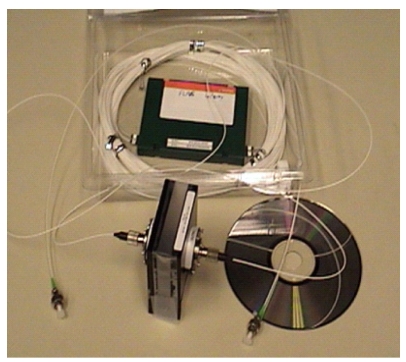
FLRD methane sensor using a U-bracket as an air-gap (gas cell).

The first report on fiber loop ringdown spectroscopy was published in 2001 [52]. Stewart *et al.* introduced an optical fiber loop with a length of several tens of meters with a 5 cm open path micro-optical gas cell for gas phase absorption measurements [52,53]. Simplified versions of fiber loop ringdown devices were introduced by both Lehmann [54,116] and Loock [55,117] groups later on. Tarsa *et al.* [54] reported their study on an optical fiber resonator for spectroscopic measurements in which the sensor head was made of a section of tapered fiber in the loop and evanescent field absorption was detected. A small volume of liquid sample was detected by this type of FLRD technique. They also demonstrated the detection of a single bio cell adsorption event [118]. Loock group [55-57] reported the detection of a small volume of dye solutions by introducing a micro-air gap into a section of fiber in the fiber loop. They demonstrated a detection limit of ∼10^–10^ mol dye solution using both a cw laser and a pulsed laser [57]. The same group further advanced the FLRD technique by introducing a phase-shift measurement, which greatly improved the data acquisition rate to close to real-time (10–100 ms) [70]. This technique has been demonstrated to be suitable for low cost, real-time, and online detection of capillary electrophoresis with a detection limit at micromole concentration levels. Using flow injections, the device can detect a series of solution samples at different concentrations. The demonstrated detection limit is 5.3 × 10^–12^ mol samples in a 530 pL (10^–12^ liter) volume. A minimum fractional absorption of 1.6 cm^–1^ for an absorption path-length of 30 μm, which corresponds to a flow concentration of 10 μM, has been demonstrated by using the FLRD technique with a fast gain switch diode laser [69-71]. Very recently, the same group has demonstrated detections of volatile organic compounds using the phase-shift FLRD technique combined with the functionally-designed polymer coating of a LPG [119]. In that work, the LPG was coated with a specialized polydimethylsiloxane polymer, which had a refractive index matched to the cladding material and was capable of extracting analytes of interest, e.g., xylene and cyclohexane, into the polymer matrix. Thus, a change in the optical transmission loss resulting from a wavelength shift of the LPG's spectral bandwidth curve was detected by the FLRD device. Xylene and cyclohexane vapors in different concentrations were detected and a detection limit of 300 ppm of xylene vapor was achieved. This work demonstrates the promise of FLRD for chemical sensing and the versatility of FLRD for incorporating a variety of sensing mechanisms into the ringdown sensing platform. In an early study, Vogler *et al.* developed a FLRD device and demonstrated measurements of the diffusion coefficient of hydrogen on silicon by monitoring the absorption of the OH radicals in the NIR region [112]. Wang *et al.* demonstrated a FLRD-based methane sensor using a U-bracket with a 2 cm air-gap as the absorption gas cell (Figure 3), and the real-time, on-line detection limit of CH_4_ was 5% [113].

### 4.2. FLRD Physical Sensors

**Figure 4.**
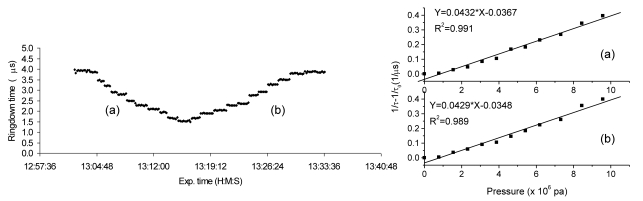
FLRD pressure sensor's response. (Left) gradually load (a) and unload pressures; (Right) good linearity of the sensor's response to pressures (Reproduction permission from Optical Society of America [60]).

Due to the ringdown enhanced detection sensitivity, high speed of measurement, and low cost for instrumentation, FLRD-based sensors have rapidly gone beyond chemical sensing to physical sensing. In 2004, Wang *et al.* demonstrated the FLRD technique for the development of pressure and force sensors [59,60,120]. A section of bare single mode fiber with a length of 1 cm was used as the sensor head. The sensing principle is primarily based on the fact that micro mechanical deformation of the fiber drastically increases optical loss in the fiber loop [59,60,111]. The sensor showed repeatable response and good reversibility to pressure changes, as shown in Figure 4 (left). Each step in Figure 4 contained many data points which were collected repeatedly at one pressure. The sensor's response to changes in pressure was less than one second. By converting the changes in ringdown time to optical losses, the sensor's response to pressure change had good linearity, as shown in Figure 4 (right). By using different configurations of the sensor heads, different sensing ranges of pressure and/or force can be achieved by the FLRD pressure/force sensor.

Using a similar FLRD approach, Wang *et al.* introduced an optical fiber Bragg grating (FBG) into the loop as the sensing element to develop FBG-FLRD temperature sensors [61-63,121]. Since the Bragg wavelength of a FBG is temperature dependent, changes in the temperature in the sensor head (FBG) are related to corresponding optical losses of the laser beam through the FBG. Different optical losses due to the shift of the FBG curve, resulting from the temperature change in the FBG, are detected by measuring the ringdown time. One of the advantages of the fiber FBG-FLRD temperature sensors is high temperature accuracy, which is not limited by the bandwidth of a FBG and the spectral resolution of an OSA. In that work [63], an accuracy of 0.06 °C was demonstrated in the temperature range of 92–114 °C.

Based on the micro-bending mechanism, which explains the induced optical losses due to fiber stain, a fiber loop ringdown strain sensor was demonstrated by Tarsa *et al* [64]. Very recently, a long period grating was introduced into a fiber loop and fiber strain sensing was reported by Ni *et al.* [65]. Since fiber loop ringdown time is a function of several parameters of a fiber loop device, including refractive index, therefore, FLRD technique can measure fiber refractive index with high sensitivity [60]. Fiber ringdown index sensor was also investigated recently [67].

FLRD sensors are still in their infancy. With its universally applicable sensing scheme and attractive application features, many FLRD sensors should be expected to come [71]. Table 3 lists the physical and chemical FLRD-based sensors which have been reported as of the writing of this article.

## 5. Perspectives on the Development of Multi-Function Fiber Optic Sensor Platforms Using FLRD

A multiple function sensor platform may include a light source, fiber loops, and a detector. The sensing scheme will be based on the FLRD technique. By adding functionally-designed FLRD sensor units (e.g., P, T, strain, gas concentration sensors) into the sensor platform, a multi-functional sensor system can be built through sensor multiplexing/integration. The high performance of the sensor system (high sensitivity, high speed of detection, and no adverse impact from intensity fluctuations of the light source) gains from the nature of FLRD. The uniform time-domain FLRD sensing scheme offers unique advantages for sensorsapos; multiplexing, data transfer/processing, and low system costs.

### 5.1. Theoretical Considerations of Multi-Function, High Performance, Fiber Optical Sensor Platforms

**High sensitivity** The detection sensitivity is often characterized by the minimum detectable optical loss. Rearranging Equation (4), we have:

5(a) $$B=\frac{t_{r}}{\tau_{0}}\frac{\Delta \tau }{\tau }=\frac{1}{m}\frac{\Delta \tau }{\Delta \tau }$$

5(b) $$\left(\Delta \tau =\tau_{0}-\tau \right)$$

where *t_r_* is the round trip time of the laser pulse in the fiber loop, and *m* is the number of round trips. Therefore, the minimum detectable optical loss *B_min_*, which is defined as the 1-σ detection limit, is given by:

(6) $$B_{\min}=\frac{1}{m}\frac{\Delta \sigma_{\tau }}{\tau }$$

where Δ*σ_τ_* is the 1-σ standard deviation of the ringdown time. Δ*σ_τ_*/τ can be experimentally achieved at the level of ∼10^–3^ [59-63], which is a typical level of the minimum detectable *ΔI*/*I_0_* in a conventional intensity-based sensing scheme (although good absorbance spectrometers can measure fractional intensity changes down to 10^–5^ in some cases). Therefore, if a conventional intensity-based fiber optic sensor has a detection limit *B*, a FLRD optic sensor will have a detection limit *B*/*m*, thus improving the detection sensitivity by a factor of *m*. Furthermore, if the intensity of the light source fluctuates, e.g., *I_0_* ± 0.2%, the detection sensitivity of the intensity-based fiber optic sensor is significantly affected; yet, the detection sensitivity of the FLRD optic sensor is not affected.

For example, assume a fiber loop sensor unit consists of 100 m single mode silicon fiber with a refractive index of 1.464, as illustrated in Figure 2(a). The coupling ratios at points 1 and 2 are both 0.1/99.9 (0.0043 dB loss). The absorption loss rate of the fiber is ∼0.3 dB/km at 1,550–1,650 nm. Therefore, the total optical losses of the laser pulse traveling in one round trip in the fiber loop will be 0.0386 dB, corresponding to 0.85% optical loss. From Equation 3(a) in Section 3, the ringdown time would be 57.4 microseconds (μs), and the round trip time, *t_r_*, would be 488 nanoseconds (ns). That means the light pulse travels the loop 118 times during one ringdown time. Therefore, if we use this FLRD sensor to measure an optical loss due to a change in, e.g., P, T, strain, or gas concentration, the detection sensitivity is ∼118 times (or two orders of magnitude) better than that obtained by a conventional intensity-based sensor. Therefore, the detectivity of FLRD is enhanced by multiple rounds of interaction in the sensor. In another words, e.g., 1,000 round trips in a ringdown time means 1,000-fold enhancement of the detection sensitivity.

**Fast response**. A typical ringdown event is on the order of μs, as shown in the above example. It has been widely demonstrated in the literature that one ringdown data point, e.g., a gas concentration, P or T, can be readily obtained within a second even when hundreds of ringdown events are averaged to improve signal-to-noise ratio in the data processing.

**High accuracy**. The measurement accuracy of the FLRD technique can be seen in a FLRD gas sensor, for example. If the gas absorption is responsible for the optical loss, *B*, in Equation(4), then we can determine the measurement uncertainty of the gas concentration from

(7) $$(\frac{1}{\tau }-\frac{1}{\tau_{0}})=\frac{c}{nL}B=\frac{c}{nL}\sigma sl$$

where *σ* (cm^2^/molecule) is the absorption cross-section of the gas at a particular frequency, *l* (cm) is the laser path through the gas, and *s* is the gas concentration (molecules/cm^3^). Since the fiber length *L* and the laser path-length *l* in Equation (7) can be accurately determined, the measurement uncertainty of gas concentration *s* is mainly determined by the ringdown time baseline stability, *Δτ*/*τ*, for a given absorption cross-section. As previously discussed, the baseline stability can be typically on the order of 0.1%. Therefore, with a know absorption cross-section at a particular frequency, FLRD fiber optic sensors can have a measurement accuracy of 0.1% of the full-scale reading. Note that absorption cross-sections documented in the literature or determined in experiments are often not 100% accurate; they typically have an error on the order of 1%–5% that is significantly larger than the ringdown baseline stability, 0.1%. Therefore, measurement accuracy of a FLRD gas sensor is ultimately determined by the accuracy of the absorption cross-section.

**Configuration of the sensor platform**. A sensor platform's configuration can be developed based on the following facts: 1) FLRD measures time to detect a quantity, 2) the time division multiplexing (TDM) and Micro-Electro-Mechanical Systems (MEMS) are established techniques used in signal processing and fiber optic network multiplexing, and 3) a FLRD decay process is on the order of μs and a light pulse experiences many round trips during one ringdown time, e.g., 118 rounds in the previous example. These three factors enable the sensing signals from multiple sensor units in a sensor platform in a serial configuration to be precisely coupled and decoupled using the TDM technique with a temporal resolution up to 0.1 ns. For instance, in the above example, if a fast data acquisition card has a time resolution of 0.1 ns or better, 4,880 signals from 4,880 sensor units can be precisely sequenced and timed within the round trip time, 488 ns. On the other hand, if MEMS are used to configure a sensor platform in a parallel configuration, the fast ringdown event (e.g., 57.4 μs in the above example) and high switching frequency of MEMS (e.g., kHz) enable more than 1000 FLRD sensor units designed for sensing different quantities to be multiplexed into a single sensor platform. In additional to the timing issues, laser power distribution also affects the maximum number of sensor units to be multiplexed. For instance, if a laser diode output is 20 mW, after 4880 consecutive injections of the laser power into 4,880 fiber loops (e.g., in a serial configuration) at a rate of 0.1% per loop, the light intensity to be injected into the 4,880^th^ loop will be only 20 × (1 – 0.001)^4,880^ mW = 0.15 mW, but it is still enough to power a FLRD sensor unit.

### 5.2. Design Perspectives of Individual FLRD Sensor Units Sensing Chemical and Physical Quantities

**Figure 6.**
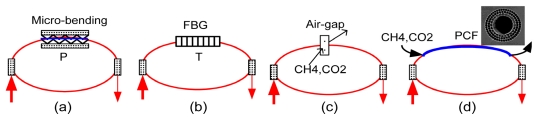
Configurations of different sensor heads.

Figure 6 illustrates four different configurations of the sensor heads, as an example, for detecting P, T, and gas concentration. For sensing P, a section of bare SMF (without plastic jacket) is typically embedded in a micro-bending platform to form the sensor head (Note that *F* = *PS*, with a known surface area *S* of the platform, the P sensor also senses the force applied). The sensitivity of FLRD pressure sensors depicted in Figure 6(a) can be characterized by the minimum detectable pressure change per ringdown time change (s), Pa/s. Wavelength selections of the FBG-FLRD temperature sensors are determined by the Bragg wavelength of the FBG which is used as the sensing element. Different from the current FBG-OSA temperature sensors, in which an OSA is used to measure a shift of the peak wavelength of the FBG, the FBG-FLRD temperature sensors measure the optical transmission losses of the laser transmitting through the FBG in terms of ringdown times, as illustrated in Figure 6(b). A narrow bandwidth FBG (with a narrow wing in each side of the bandwidth curve) can provide high sensitivity (because a small shift drastically changes transmission rate at a given wavelength). FBGs, long period gratings (LPGs), and linear fiber gratings (LFGs) can be employed to form temperature sensor heads to achieve different sensing properties, such as sensitivity, temperature measuring range, response linearity, etc. [51,124-126]. The sensitivity of FLRD temperature sensors can be characterized by the minimum detectable temperature change per ringdown time change, °C/s.

For CH_4_ and CO_2_ sensors, two NIR telecommunications laser diodes can be used for the detection of CH_4_ and CO_2_ at 1,651 nm and 1,572 nm. Wavelength selections should be based on a combined consideration of sensitivity and possible spectral interferences, and they can be characterized based on a spectral simulation using HITRAN 96 [127,135]. For the sensor heads, a U-shape air-gap bracket [as seen in Figures 3 and 6(c)] [113] with ultra-low insertion loss (e.g., <0.1 dB) and high thermal and mechanical stability can be acquired. Gas samples can be directly flowing through or static in the air-gap. The sensing mechanism is based on Beer's Law. Therefore, as compared with the conventional single-pass absorption, the detection sensitivity will be enhanced by the presence of multiple round trips as detailed in Section 3. Due to the limited length of the air gap, e.g., 1 cm (longer air gaps, higher sensitivities, but also larger optical losses), the detection limit of this configuration is typically at the levels of a few percent. The air-gap configuration is advantageous for chemical identification (note that with the same path-length and spectral region, CH_4_ is not detectable by the single-pass absorption scheme). In order to further enhance the detection sensitivity, a photonic crystal fiber (PCF) with air holes in the fiber core [Figure 6(d)] can be fabricated into a fiber loop with low optical insertion loss. Current technology allows for the lowest insertion loss in the connection of SMF with PCF to be <0.3 dB. Using a section of PCF as a gas cell in the fiber loop is desirable. In this way, a long portion of the fiber loop can be filled with gas samples. The detection sensitivity for the same species at the same absorption wavelength would be doubly enhanced by both the nature of the multiple round trips of the laser pulse in the loop and the long path-length of the laser pulse in the sample in each round trip (e.g., from 1 cm of the air-gap to 1 m of air-hole PCF per round trip), this directly results in an additional sensitivity enhancement by 100-fold (note that with a longer PCF, the gas diffusion time will be longer).

### 5.3. Design Perspectives of the Sensor Platforms in a Serial Configuration

**Figure 7.**
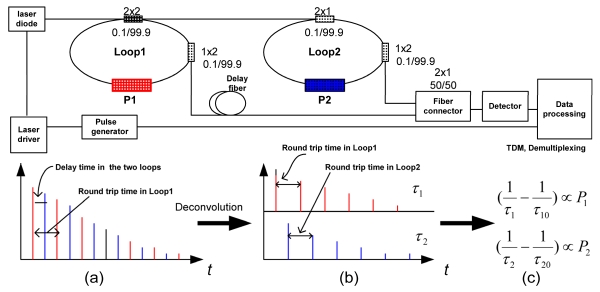
Proposed concept of the sensor platform in a serial configuration. A platform consisting of two sensor units is used to illustrate the concept. (a) Signals from the two sensor units are coupled; Red: from Loop1; blue: from Loop2. (b) Decoupled signals from determination of P1 and P2.

Figure 7 illustrates a design perspective of a FLRD sensor platform in a serial configuration (bus configuration in networking topology) [94]. The concept of the integration and control of the two sensor units in the serial configuration is based on the TDM technique used in digital signal processing and has been demonstrated recently in a double fiber loop ringdown system by Li *et al.* [24,123,128-133]. A laser pulse of intensity I_0_ from a diode laser operating in the telecommunications C-band, e.g., at 1,550 nm, is injected into the fiber coupler. A 0.1% of I_0_ is coupled into Loop1 through the fiber coupler. The rest of the light (99.9% I_0_) will be used as the input light pulse for Loop2, where 0.1% of the 99.9% I_0_ will be coupled into Loop2. Each loop will have individual ringdown events, which yield different ringdown times (determined by length of the loop, insertion losses, etc.). The detector (photodiode) observes a coupled signal from both of the loops as illustrated in Figure 7(a). The TDM technique in the data processing portion will be used to demultiplex the coupled ringdown signal into two individual ringdown decays, each of which yields a separate ringdown time [122,123]. The time delay between the two ringdown events occur in the two loops is adjusted by the length of the delay fiber. In this configuration, the time sequence determines the location of each sensor unit (to distinguishing which is which) and the change in ringdown time in each loop relates to a change in the magnitude of a measurand in each unit.

Each ringdown decay waveform monitored by the detector will be input to an analog to digital (A/D) converter, digitized into, e.g., 1,000, data points, and transferred to a computer in the electronic module for processing. The data points will be fitted into a single exponential decay waveform to obtain the ringdown time by first taking logarithm of the voltage reported by the photodetector at each sample point and then using linear regression to determine the slope [127]. Fast A/D conversion can have a time resolution of ns. Different combination modes, e.g., two P sensors, one P sensor and one T sensor, and two T sensors, can be achieved. In principle, more sensor units can be added to the platform to form a multi-functional sensor system. The maximum number of sensor units in the platform is determined by the minimum resolvable time delay between two adjacent loops and by the round trip time, as discussed in Section 3. Potential applications of this serial configuration include situations that necessitate two (or more) sensors to be deployed in different locations with a specifically separate distance, e.g., force monitoring along a bridge, leakage detection along a pipeline, etc. It must be noted that by rearranging the fiber loops, sensor units can be also deployed in a parallel fashion while the multiplexing and decoupling of the signal is still based on the TDM technique.

### 5.4. Design Perspectives of the Sensor Platforms in a Parallel Configuration

**Figure 8.**
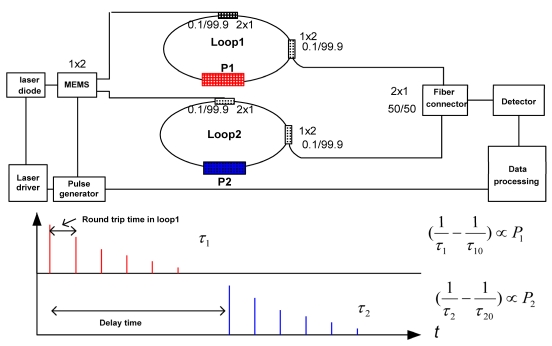
Proposed concept of the sensor platform in a parallel configuration. A platform consisting of two sensor units is used to illustrate the concept.

Figure 8 illustrates a speculative design of a parallel configuration of the sensor platform. The multiplexing and control can be achieved by using the MEMS optical switching technique [130]. Experimental issues in this design will include the MEMS optical switching frequency and associated electrical and optical noises, which affect ringdown baseline noise. Data deconvolution algorithms in terms of accuracy, data processing time, intelligent control, and the capability of networking will be different from the ones in the serial configuration.

In this parallel configuration, signals from the two sensors are decoupled. The sensor system alternatively measures P1 and P2 with a time delay between each measurement set by the delay time which triggers the MEMS. Current MEMS can have 1 × 32 channels or higher with switching frequencies up to MHz. Selection of switching frequency should take into consideration the duration of each ringdown measurement event. The inverse of the switching frequency of the MEMS must be greater than ringdown decay time so that each ringdown event can be completely measured (data is transmitted and ringdown time is derived). For instance, if a ringdown event is on the order of μs, then the highest switching frequency of can only be ∼100 kHz. This assumes use of a fast A/D converter and data transmission processing [127]. The maximum number of sensor units in a platform is not restricted by the limit of the TDM mentioned above. This configuration is to some extent advantageous over the serial configuration in signal processing since the signals from different loops are not coupled. A hybrid configuration (serial connections in each of the parallel branches) can be also achieved.

### 5.5. Perspective on an Integration of a Multi-Functional Sensor System Based on FLRD Sensor Platforms

**Figure 9.**
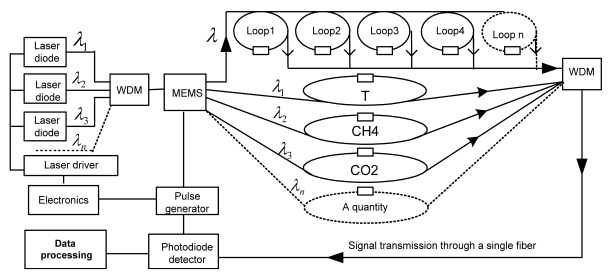
Proposed multi-functional fiber optic sensor system formed by adding FLRD sensor units to the sensor platform, which consists of a light source, a detector, and FLRD sensor units.

Figure 9 shows the architecture of a multi-functional sensor system formed by adding sensor units to the sensor platform. The top four sensors (Loop1 – Loop4) detect the same quantity, e.g., pressure (P), at different locations; the bottom four sensor units detect T, CH_4_, and CO_2_ at the same location (can be in different locations, too). All of the sensing signals from each individual sensor unit are fused and transmitted uniformly through a single fiber linked to the detector that observes a coupled ringdown decay. Location of each sensor will be determined by the time sequence as discussed in the preceding sections. Each quantity, such as P, T, and gas concentrations, will be determined by individual ringdown times, which are obtained from deconvolution of the coupled ringdown decay. Laser diodes operating at different wavelengths are multiplexed by using WDM [24,127,136,137], and the MEMS selectively controls the laser beam with the needed wavelengths to be injected into the fiber loops (or loop branches) for detection of different quantities. The entire sensor system uses a single detector to monitor a single coupled ringdown decay and detects the four quantities simultaneously. Although FLRD chemical sensors also use spectral fingerprints to identify gas species and determine absolute gas concentrations based on absorption, this new sensor platform cannot be achieved by the conventional intensity-based sensing scheme. For instance, a FBG-OSA temperature sensor measures wavelength shift, *I*-Δλ, and a conventional fiber gas sensor measures *ΔI*/*I_0_*. Configuring these different detection schemes into a single sensor platform would be practically impossible, unless an array of terminal detection equipment, such as OSAs, photodiodes, etc. are bundled together and signal from each sensor unit is detected separately. In that case, sensor system (networking) costs, response time, and data transmission/processing efficiency would become significant issues.

Theoretically, many more sensor units can be added to the platform, as illustrated by the n^th^ sensor unit marked by the dashed line in the figure. In principle, such a sensor platform can form a sensor system that has *n* sensor units for detecting ≤ *n* parameters at *n* different locations; and n is determined by the round trip time of the fiber loop and the time resolution of the data acquisition card, as discussed in Sections 3 and 5.1. Various sensing mechanisms can be directly adopted to configure sensor heads to form different FLRD sensor units for measurements of different parameters, such as stress, strain, vibration, and chemical species. Figure 9 just shows one type of configuration of the sensor platform. Other configurations of the sensor platform can also be speculated. Different configurations certainly have their own advantages and limitations. For instance, potential optical interference between two adjacent laser beams may be generated in the serial configuration [122]. Influence of the interference effect on signal-to-noise ratio and data processing should be a challenging issue. The parallel configuration may be advantageous with regard to this point; however, the data sampling rates will be relatively lower than that in the serial configuration due to the alternative measurement approach. Since the signals from the different units are decoupled in the parallel configuration, this feature may results in an easier and faster data acquisition. Although FLRD is insensitive to power fluctuations and requires minimal laser power, effect of laser power on the networking of the sensor system needs to be also considered.

## 6. Conclusions

FLRD is relatively new to the spectroscopy and gas sensing communities, and its potential for development of new generation, multi-function, sensing platforms has not been discussed much. This paper gives a brief review on the current FRLD-based fiber optic sensors with an emphasis on potential of development of new generation, multi-function, fiber optic sensor platforms using the FLRD technique. Several speculative examples are given to illustrate the new concept, which may help advance the field of sensing science and technology beyond the single function (quantity), single location sensing limited by the conventional sensing scheme to the simultaneous multi-function, multi-location sensing using a new time-domain FLRD sensing platform.

## Figures and Tables

**Figure 5.**
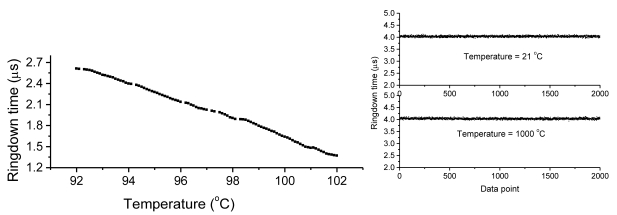
FLRD temperature sensors. (Left) FBG as the sensing element showing high precision; (Right) showing high baseline stability at low and high temperatures (Reproduction permission from Institute of Physics [62]).

**Table 1.** Current FOS in terms of sensing functionalities, sensing mechanisms, and sensing schemes.

**Sensing function**	**Sensing mechanism**	**Sensing scheme**	**Sensor head configuration**
Gas concentration /identification	Beer's Law based direction absorption, evanescent wave absorption	Intensity vs. frequency (spectrometer based)	Air-gap, side-polished cladding, tapered bare fiber, U-bend
Gas concentration /identification	Fluorescence	Intensity vs. frequency (spectrometer based), intensity vs. time (fluorescence life time based)	Unmodified fibers, decladded fibers, doped cladding, excitation through central core and emission through outer fiber, bifurcated fiber bundle, U-bend decladded, tip based, tip with active cladding, etched tip, modified end-face
Chemical/ biochemical agents	Agent-induced changes of refractive indices, surface plasmon resonance	Intensity vs. wavelength (spectrometer-based), imaging of fluorescence, scattering	Agent selective polymers plus long period gratings (LPGs)in photonic crystal fiber (PCF), various forms of metal/dielectric interface
Pressure	Mechanical deformation	Interferometric spectral patterns	FPI, FBG, tapered bare fiber
Temperature	Thermal expansion	Reflected spectral patterns	FBG, LPG, FPI, U-bend, doped composition fiber for high T
Stress/strain	Mechanical deformation	Reflected spectral patterns	FBG, LPG, FPI, U-bend, tapered bare fiber
Vibration	Mechanical deformation	Reflective configuration due to displacement of transducer	FBG, FPI

**Table 2.** A comparison of the FBG-FLRD scheme with the FBG-OSA scheme (Both use a SMF bare FBG as the sensing element and the thermal sensitivity is ∼0.01 nm/°C) [62].

**Features**	**FBG-FLRD scheme**	**FBG-OSA scheme**
Measuring domain	Time	Frequency
Sensing scheme	Time constants (τ)	Spectral shifts (Δ*λ*)
Measurement resolution	0.18 °C (based on 3-σ), not limited by detector	2 °C, limited by OSA's spectral resolution
Detection sensitivity (if same sensing mechanism is used)	Enhanced by the multiple-round trip effect	Single pass (single interaction)
Influenced by intensity fluctuations of light source	No	Yes (when the spectral shift is close to the full width at half maximum of the FBG's bandwidth curve)
Speed of detection	High measuring speed (up to kHz)	Low
Cost of detector (or terminal equipment)	Photodetector (PD) (∼$240)	OSA with a resolution of ±0.02 nm (∼$5,000–8,000)
Potential for sensor multiplexing	Promising	Limited by power losses, power fluctuations, ASE noise

**Table 3.** Reported FLRD-based FOS.

**Sensing function**	**Sensing mechanism**	**Sensor head configuration**
Gas concentration	Beer's Law based direction absorption, evanescent wave absorption	Air-gap, tapped bare fiber, and chemically-coated LPGs [52-54,58,113,118,119]
Microfluidics	Direction absorption, evanescent wave absorption	Air-gap, capillary [56,57,68]
Single molecular cell	Light scattering	Tapered fiber [118]
Biomolecules (protein analysis)	Absorption	Capillary-fiber interface [69]
Pressure/force	Mechanical deformation	Bare single mode fiber (SMF) [59,60,120]
Temperature	Thermal expansion	FBG, LPG [61-63,121,122]
Stress/strain	Mechanical deformation	FBG, bare SMF [64-66]
Refractive index	Light traveling speed	LPG, bare SMF [60,67]

## Abbreviations

A/D: analogy to digital
ASE: amplified spontaneous emissions
CRD: cavity ringdown
CRDS: cavity ringdown spectroscopy
FBG: fiber Bragg grating
FBG-FLRD: fiber Bragg grating-fiber loop ringdown
FBG-OSA: fiber Bragg grating-optical spectral Analyzer
FLRD: fiber loop ringdown
FOS: fiber optic sensor(s)
FPI: Fabry-Perot interferometric
LPG-FLRD: long period grating-fiber loop ringdown
LPG: long period grating
MEMS: micro-electro-mechanical systems
NIR: near infrared
OSA: optical spectral analyzer
PCF: photonic crystal fiber
P: pressure
SMF: single mode fiber
TDM: time division multiplexing
T: temperature
WDM: wavelength division multiplexing
